# Efficacy of Transcranial Direct Current Stimulation and Photobiomodulation in Improving Cognitive Abilities for Alzheimer’s Disease: A Systematic Review

**DOI:** 10.3390/jcm14051766

**Published:** 2025-03-06

**Authors:** Monica Cornea, Bogdan Ioan Vintilă, Mihaela Bucuța, Laura Ștef, Claudia Elena Anghel, Andreea Maria Grama, Andrei Lomnasan, Andreea Angela Stetiu, Adrian Boicean, Mihai Sava, Lucian Constantin Paziuc, Mihnea Costin Manea, Andrian Tîbîrnă, Ciprian-Ionuț Băcilă

**Affiliations:** 1 “Dr. Gheorghe Preda” Clinical Psychiatry Hospital of Sibiu, 550082 Sibiu, Romania; moni.sipos@ymail.com (M.C.); claudia.anghel@ulbsibiu.ro (C.E.A.); andreeamariaps@gmail.com (A.M.G.); andreilomnasan96@gmail.com (A.L.); ciprian.bacila@ulbsibiu.ro (C.-I.B.); 2 Faculty of Medicine, Lucian Blaga University of Sibiu, 550169 Sibiu, Romania; laura.stef@ulbsibiu.ro (L.Ș.); andreea.stetiu@ulbsibiu.ro (A.A.S.); adrian.boicean@ulbsibiu.ro (A.B.); mihai.sava@ulbsibiu.ro (M.S.); 3 County Clinical Emergency Hospital of Sibiu, 550245 Sibiu, Romania; 4 Neuroscience Scientific Research Collective, 550082 Sibiu, Romania; 5 Faculty of Social Sciences and Humanities, Lucian Blaga University of Sibiu, 550024 Sibiu, Romania; 6 Campulung Moldovenesc Psychiatric Hospital, Trandafirilor Street 2, 725100 Câmpulung Moldovenesc, Romania; paziuc.lucian@yahoo.com; 7 “Prof. Dr. Alexandru Obregia” Clinical Hospital of Psychiatry, 041914 Bucharest, Romania; mihnea.manea@umfcd.ro (M.C.M.); atibirna@yahoo.com (A.T.); 8 Faculty of Medicine, Carol Davila University of Medicine and Pharmacy, 8 Eroii Sanitari Bvd, 050474 Bucharest, Romania

**Keywords:** tDCS, photobiomodulation, efficacy, safety, Alzheimer

## Abstract

**Background:** Due to the increasing global prevalence of Alzheimer’s dementia (AD), neuromodulation techniques such as transcranial direct current stimulation (tDCS) and photobiomodulation (PBM) are considered potential complementary therapies. Objective: We assessed the efficacy and safety of tDCS and PBM and their potential to enhance cognitive functions in individuals with AD. **Methods:** This review primarily examined studies designed to evaluate the efficacy, followed by an assessment of the safety of tDCS and PBM for people with AD. The databases searched were PubMed, Scopus, and Web of Science Core Collection, resulting in 17 published randomized and controlled trials. References were screened over 5 years (2020–2024). The research design used PRISMA guidelines. **Results:** Fourteen studies were considered for tDCS, and the current literature supports efficacy and safety at an amperage of 2 mA, with electrodes placed on the dorsolateral prefrontal cortex (DLPFC). Three studies were included for PBM. The heterogeneity of these study measures made them unsuitable for combined efficacy analysis, and they did not provide a safety evaluation. **Conclusions:** Despite differences in efficacy assessments, tDCS and PBM improved cognitive abilities. There is an urgent need to standardize metrics for evaluating efficacy and safety, particularly for PBM. Future research is encouraged.

## 1. Introduction

Alzheimer’s disease (AD) is a neurodegenerative disorder affecting millions of people and their caregivers all over the world, and it is considered to be the most common cause of dementia AD [1]. The etiology and pathophysiology of AD are more complex even though intracellular neurofibrillary tangles (NFTs) and extracellular amyloid-beta (Aβ) plaques are thought to be hallmarks of the disease [2]. For people diagnosed with AD, cognitive impairment is progressive and affects problem-solving, language, and other cognitive functions [3,4].

The research performed in the last years indicates that mild cognitive impairment (MCI) is a potential phase in which normal cognition might become mild dementia due to the underlying AD [5,6,7]. Therefore, preventing or delaying the onset of the final stage of AD may be possible if we understand the factors that can influence pathology in the early stages [8,9]. For many years, it was considered that the etiology of AD is the neurotoxic effects of amyloid aggregates [10]. Despite theory, amyloid plaques have been found in individuals with normal cognitive functions [11]. Also, the correlation between the density of amyloid plaques and the severity of cognitive impairment is considered to be unsatisfactory [12]. Another theory that attempts to explain the etiology of AD is the mitochondrial cascade hypothesis, which states that both hereditary and environmental variables could alter mitochondrial function. Patients with AD are considered to have abnormal mitochondrial metabolism characterized by reduced oxidative phosphorylation, diminished ATP production, and elevated levels of reactive oxygen species [11,13,14,15,16]. A neuro-energetic hypothesis suggests that a reduction in glucose levels crossing the blood–brain barrier may induce low energy stress in the central nervous system (CNS), potentially resulting in the accumulation of amyloid beta and tau proteins [17].

Despite an enormous scientific effort focusing on this AD, there are presently few disease-modifying medicines available. Cholinesterase inhibitors and glutamate receptor antagonists are the primary anti-dementia medications used to prevent the progression of AD [18]. Currently, anti-amyloid monoclonal antibodies have received approval for treating early Alzheimer’s disease patients [19]. The investigation of antioxidants yielded inconclusive outcomes [20,21].

Although treatment for AD is considered by many clinicians, mainly pharmacological, other non-pharmacological therapies could benefit people with AD. Cognitive stimulation is an intervention used to maintain cognitive functions in people with AD, and this approach may prove particularly beneficial for individuals with moderate severity of the disease [22]. Other non-pharmacological approaches that are deemed to bring functional and social benefits to people diagnosed with AD are non-invasive brain stimulation and modulation [23].

NIBS (non-invasive brain stimulation) techniques are neuromodulating methods known for impacting cognition, emotions, and behavior through alterations in neuronal plasticity. Also, they are reported to alter brain networks, and this may be beneficial in enhancing cognition in neurodegenerative disorders [23,24,25,26]. Understanding NIBS-induced network effects may optimize the use of NIBS therapy for neurodegenerative diseases caused by progressive neural network disruption. AD is typically characterized by disruptions in memory-related networks, including the default mode and limbic networks [27]. These large-scale neuronal networks, which serve as intermediaries between the molecular pathology of neurodegenerative conditions and their clinical symptoms, may be influenced by brain stimulation [28].

tDCS is a NIBS technique that has been used in clinical research since 1998 [29]. This method became a tool for neuromodulation in various regions of the human cortex due to its ability to promote neuroplasticity. This function was discovered through its investigation of the effects on the motor cortex. The mechanism of tDCS consists of anode–cathode activity: the anodal electrode enhances neuronal excitability, while the cathodal electrode depolarizes the resting potential. These modifications in neural sensitivity can be observed at the behavioral level and have been demonstrated to influence the subsequent learning and memory consolidation of tasks that are presented during stimulation [30,31,32]. Nevertheless, its impact on more generalized learning beyond the task that is specifically asked for is a relatively unexplored area [33].

Numerous reviews and meta-analyses have meticulously examined the effects of NIBS in neurodegenerative diseases and have proposed that tDCS may have a beneficial influence on cognition in dementias despite the limitations of this approach [34,35,36,37,38,39].

Photobiomodulation (PBM) is an additional neuromodulation technology used for brain stimulation [40]. It uses non-retinal exposure to wavelengths (visible red light or Near-Infrared Radiation (NIR)) to stimulate, heal, or restore affected cells and tissue. PBM modulates brain activity in two ways: by enhancing mitochondrial ATP production and increasing regional cerebral blood flow (rCBF) [41,42]. Therefore, it is essential to understand its role in the treatment and prevention of AD, as it is associated with hypometabolism in specific brain regions and mitochondrial dysfunction. Studies on various diseases have shown PBM’s safety and effectiveness in people, establishing PBM as a possible therapy approach [42,43,44,45,46].

The aim of this review is to explore the efficacy of using tDCS or PBM as therapy for improving the cognitive abilities (memory, praxis, and language) of people affected by AD.

## 2. Materials and Methods

### 2.1. Literature Search

For this review, the following primary research question was stated: what is known from the literature about the efficacy of applying tDCS, or PBM, for improving the cognitive abilities (memory, praxis, and language) of people affected by Alzheimer’s disease?

Three databases—PubMed, Scopus, and the Web of Science Core Collection—were searched to investigate various sources. Over five years, the following terms were used to search in all three databases: “tDCS efficacy Alzheimer”, “tDCS cognitive function Alzheimer”, “photobiomodulation efficacy Alzheimer”, and “photobiomodulation cognitive function Alzheimer”.

This research design considered 451 hits to be screened (PubMed = 196, Scopus = 133, and Web of Science Core Collection = 122). Two authors, CM and BC, conducted the literature search and article screening process. References were screened over 5 years (2020–2024). This research design was constructed considering systematic review and meta-analyses (PRISMA) guidelines [47].

### 2.2. Inclusion and Exclusion Criteria

Only articles that addressed the efficacy of tDCS and PBM in improving the cognitive functions of people diagnosed with AD were considered.

The inclusion criteria were as follows:

(1) English language;
(2) Human participants;
(3) Studies that evaluated efficacy over the cognitive functions while using tDCS or PBM;
(4) Studies that evaluated safety while using tDCS or PBM;
(5) Alzheimer’s disease diagnosis.

The exclusion criteria were as follows:

(1) Simulated studies;
(2) Studies that did not evaluate the efficacy over the cognitive functions;
(3) Studies using only other NIBS (noninvasive brain stimulation techniques) as treatment;
(4) Studies on pediatric population.

Although there are other types of neuromodulation techniques, such as transcranial alternating current (tACS), transcranial random noise stimulation (tRNS), and transcutaneous spinal direct current stimulation (DCS), this research focuses only on tDCS and PBM.

### 2.3. Data Collection and Outcome Measures

After reviewing the articles collected from the database searches, we constructed a list of variables from which the data used in this research were gathered, and it included the following:

(1) Metadata (authors names, journal and publication date);
(2) Demographic data (age, sex, and number of participants);
(3) Data-collecting methods (randomized controlled trial (RCT) and side-effects evaluation);
(4) tDCS protocol (amperage used, active/sham stimulation, duration of stimulation, number of sessions performed, electrode size and location, and content of saline solution);
(5) PBM protocol (wavelength, pulse frequency, intensity, duration of stimulation, numbers of sessions performed, way to administer (transcranial, intranasal), and active/sham);
(6) Efficacy;
(7) Cognitive abilities;
(8) Reported medications used;
(9) The author’s conclusions (whether tDCS and PBM are effective and improve cognitive abilities based on their assessments).

The primary outcomes were the efficacy of tDCS and PBM in improving the cognitive functions of people diagnosed with AD. The secondary outcomes also included safety and tolerability and verification of the influence of other variables on the primary outcomes.

### 2.4. Risk of Bias

**Figure 1:**
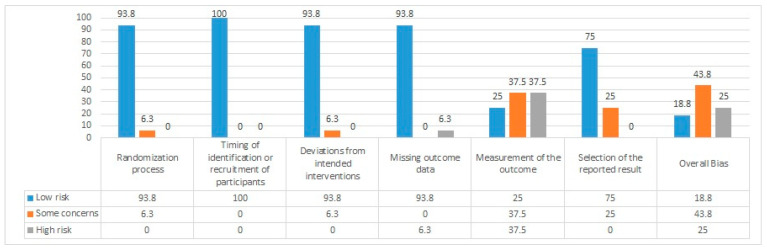
Risk of assessment bias for the included studies.

For this literature review, the Cochrane Collaboration’s tool for risk of bias in randomized trials, namely the revised tool to assess the risk of bias in randomized trials (RoB 2) [48], was used to assess the risk of bias among the included studies. This tool included six domains for bias: randomization, timing of identification/recruitment of participants, deviations from intended interventions, missing data, outcome measurement, and selection of reported results. These biases were rated as low risk, high risk, and some concerns. The risk of bias for the studies included in this review is illustrated in Figure 1.

#### Quality of Studies

Figure 1 above presents the bias risk assessments for the included studies. Overall, bias showed some concerns (43.8%).

All but one study provided a comprehensive description of the randomization technique. Similarly, this applies to the deviation from the intended intervention and the absence of outcome data domains. Most of the studies lacked transparent measurement of outcomes, resulting in a 37.5% concern and a 37.5% high risk of bias. Most of the studies in this review showed a low risk of bias regarding selecting the domain of the reported result. No studies included reported any risks associated with identifying or recruiting patients, as illustrated in Table 1.

## 3. Results

### 3.1. Literature Search

Our initial search yielded 451 references for title and abstract review. After removing all the duplicates (105 sets of duplicates), 207 abstracts were reviewed again to apply the inclusion and exclusion criteria.

**Figure 2:**
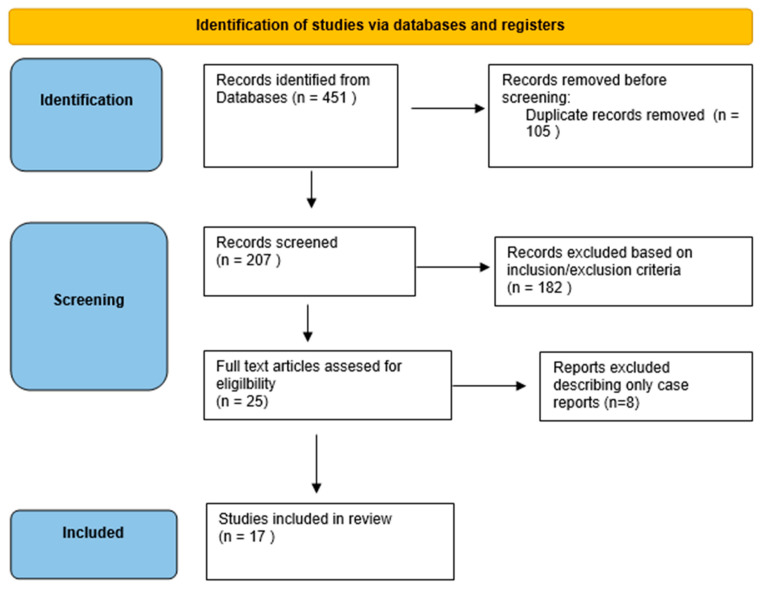
Literature-search PRISMA flow diagram.

A total of 25 articles were selected for full-text review and data extraction. BC and CM completed text reviews and data extraction. BC verified all text reviews and data extraction. Seventeen articles were chosen for this review. tDCS was used as a therapy in 14 articles, and PBM was used in 3 articles [49,50,51,52,53,54,55,56,57,58,59,60,61,62,63,64,66]. Figure 2 presents a graphical representation of the study’s screening and selection process using PRISMA guidelines [47,67].

### 3.2. Efficacy and tDCS

#### 3.2.1. Efficacy and Electrode Location

Researchers must consider the effectiveness of tDCS with electrodes positioned at various places when determining dosage. Since each potential anatomical site may correspond to a specific physiological response, testing one site or using one montage could be sufficient for concluding each potential site. Anode/cathode location depends on where the anode is placed over the ipsilesional cortex. This review has documented at least 10 unique montages corresponding to seven brain regions: left and right DLPFC (dorsolateral prefrontal cortex), Broca, Wernicke, superior and inferior parietal lobe, and superior angular gyrus. The bilateral montage was mainly used (see Table 2).

#### 3.2.2. Electrode Size, Conducting Material, and Amperage

Electrode size and conducting material influence the stimulation’s diffusivity or locality via current density, so this information was mandatory to report (see Table 1). A total of 86% (n = 12) of the present studies included precise electrode size. Most reviewed studies used the common 5 × 7 (35 cm^2^) or 5 × 5 (25 cm^2^) electrodes. Only 78% of articles (n = 9) reported the composition of their soaking solution (saline solution).

In 93% of the studies (n = 13) included in this review, the amperage used was 2 mA (see Table 1). Only Pini [59] used 1.5 mA in their research. The literature documents this value for amperage (2 mA) as effective for tDCS in treating degenerative brain disorders like AD [25].

#### 3.2.3. Efficacy and Medication

Evaluating medication usage is essential to implement tDCS in clinical settings effectively. Only five studies included in this review reported medication usage, with none detailing the dosage. Philippen [59] provided only the medication class (antidementia), and Gangemi [52,53] and Hu [54] mentioned the type of medication but not the dose. No studies reported any contraindications. In one particular study, Lane [57] detailed the medication used (sodium benzoate), including the class of drugs, type, dosage, and adverse effects. This information was pertinent as the application of sodium benzoate constituted a key parameter within the randomized study conducted. The same study also noted the use of benzodiazepines, including the maximum permitted dosage; however, it did not provide information regarding the number of patients using this medication or side effects.

#### 3.2.4. Efficacy for Specific tDCS Devices

The tDCS devices used in most studies included in this review differed (11 types). The results obtained do not appear to be influenced by the specific type of device utilized.

#### 3.2.5. Efficacy and Neuroimaging

This review identified two primary neuroimaging technologies used in the studies: electroencephalography (EEG) and magnetic resonance imaging (MRI). EEG was used in three studies [51,52,53], and MRI was used in three studies [59,60,61]. Notably, none of the studies incorporated both EEG and MRI investigations. One study used an FDG-PET scan [55].

##### EEG

In this review, 21% (n = 3) of the studies used EEG to assess changes associated with tDCS. In all of these cases, no pathological changes were observed. Andrade [51] explored quantitative EEG measures, including spectral power analysis. They found that increased high-frequency power following active tDCS and cognitive stimulation could enhance cognitive abilities in patients with AD. It was noted that the more significantly improved the ADAS-Cog, the greater the spectral power for both high and low frequencies in the active group. Additionally, Gangemi [52,53] utilized electroencephalography in his studies to assess whether tDCS could enhance cognitive functions. In his 2020 study, it was reported that tDCS improves cognitive abilities, accompanied by an increase in beta band activity and a decrease in P300 latency. In his 2021 study, the findings indicated that while tDCS can positively affect EEG patterns, the effects are likely to be significant only after a long-term intervention of eight months.

##### MRI/PET

A total of 21% (n = 3) of the studies included in this review utilized MRI to assess patients before/after receiving tDCS and its efficacy. A total of 7% (n = 1) of the studies used FDG-PET scans to evaluate patients before and after receiving tDCS and its efficacy. Jamie [55] and Rassmusen [61] also described a computational model. Using FDG-PET scans, Jamie [55] suggested that tDCS might positively affect glucose metabolism in the middle and inferior temporal regions, potentially enhancing cognitive functions. In his study, Phillipien [59] concluded that the medio-temporal atrophy score improved following tDCS application, and this improvement correlated with lower hippocampal volumes. Pini [60] utilized MRI before and after administering tDCS, focusing on brain networks affected by AD. The results indicated that tDCS increased activity in the default mode network during anodal stimulation, possibly contributing to restoring cognitive abilities. In his study, Rasmussen [61] utilized MRI scans to evaluate the effects of the tested intervention both before and after its application. The findings indicated that tDCS is effective in improving delayed memory in people diagnosed with AD.

### 3.3. Safety and tDCS

#### Safety, Tolerability, and Side Effects

In the studies included in this review, several assessment methods were utilized to evaluate the cognitive effects of applying tDCS as a treatment and to measure the outcomes, including possible side effects. In only one study, Philippen [59] described a specific cognitive side effect of tDCS sessions, such as fatigue.

Some studies included in this review used specific questionnaires, enabling patients or caregivers to assess systematic changes in task performance or self-reported outcomes, like the Mini-Mental Status Examination, Alzheimer’s Disease Assessment Scale-Cognitive Subscale, Wechsler Memory Scale, Neuropsychiatric Inventory, The Barcelona Test-Revised, Rey’s Complex Figure, The Clinical Dementia Rating, and others. None of the results of scales used in the abovementioned studies showed any decline in verbal fluency, speech, memory, or mental status after tDCS therapy was applied. Because of the heterogeneity in these study measures, they were unsuitable for a combined analysis.

In total, 43% (n = 6) of the studies reported side-effect evaluations, as can be seen in Table 3. In two studies, the authors reported using structured questionnaires and scales to study side effects. Pini [60] assessed the side effects of tDCS application using two structured questionnaires: one administered at the beginning and end of the tDCS therapy, and the other used to monitor any adverse effects that could occur within 24 h after each tDCS session. Lane [57] utilized the UKU Side-Effects Rating Scale not to evaluate the side effects of the tDCS session but to determine the adverse effects that the medication administered in the study (sodium benzoate) could produce. Providing a simple all-or-nothing statement was often standard practice when no side effects were observed.

The most common side effects described in studies that reported side effects were discomfort, pain, warmth/burning sensation, itching, and fatigue. Phillippen also reported a metallic taste. The majority of the study’s participants rated the side effects as mild. However, Philippen also indicated moderate intensity. In addition, his study described side effects in both active and sham groups [59].

### 3.4. Efficacy and PBM

#### 3.4.1. Efficacy and Method of Administration

In all the studies included in this review, PBM therapy is administered in the cranial region. Nagy [49] employed a nasal PBM device, while Razzaghi [50] used a Light-Emitting-Diode device with headsets to deliver light through the scalp. Maksimovich [64] described a transcatheter intracerebral laser PBM device, which is more invasive (it requires local anesthesia and specific treatments to prevent clotting during its use)

The materials and features used differ in all the studies included in the review, and the underlying method is inconsistent. Although the delivery methods described vary, the outcomes regarding efficacy are positive (see Table 4).

#### 3.4.2. Parameters Evaluated

The parameters considered are heterogeneous since the approach differs in all the studies in this review. The description of the parameters varies in all the studies. Razzaghi [50] utilized the following parameters to implement PBM therapy: wavelength, power density per LED, pulse frequency and duration, timing of treatment, number of sessions, beam spot size, energy density per LED, dose, and cumulative dose per week. Maksimovich utilized the following parameters: wavelength, laser output power, fiber output power, treatment session duration, beam spot diameter in the vessel, and average dose during treatment. Nagy provided limited information regarding the parameters used to implement the PBM therapy [49,64]

#### 3.4.3. Efficacy and Medication

Evaluating medication usage is important for effectively implementing PBM in clinical settings. There is a significant deficiency in medication reporting within existing studies and the wider PBM literature. In total, 66% (n = 2) reported medication usage; however, only Razzaghi [50] indicated that patients continued their antidementia medication while receiving PBM therapy. In Maksimovich’s study [64], the antidementia medication was noted as being used solely by the control group, while patients receiving PBM did not receive any antidementia medications. A total of 33% (n = 1) did not provide information regarding medication use.

#### 3.4.4. Efficacy and Neuroimaging

This review identified multiple neuroimaging technologies used in the studies included, but MRI was used in only two studies. Razzaghi [50] used MRI to assess patients before initiating PBM therapy, but there is no description of imaging conducted during or after the completion of the PBM sessions. Due to the complex and invasive PBM method, Maksimovich [64] described a broad set of imaging techniques: scintigraphy of the brain, rheoencephalography, CT, MRI, and MUGA. In the third study included in this review, Nagy [49] provided no information regarding neuroimaging.

### 3.5. Safety, Cognitive Effects, and PBM

In the studies included in this review, several assessment methods were utilized to evaluate the cognitive effects of applying PBM as a treatment and to measure the outcomes. Heterogeneous psychological scales, such as the Mini-Mental State Exam, Montreal Cognitive Assessment, Disability Assessment for Dementia, Clinical Dementia Rating, and Hamilton Anxiety and Depression Rating Scales, were used to evaluate patients or quantify the effects of PBM therapy. No study in this review provided information regarding PBM sessions’ cognitive or other side effects.

## 4. Discussion

The results overview the available tDCS and PBM efficacy research in AD patients. The numbers below represent only a fraction of the published literature on PBM and tDCS, yet they constitute a significant subset that specifically examines objective efficacy measures.

The revised tool to evaluate the risk of bias in randomized trials (RoB2) was used to assess the risk of bias for this review. Most of the “some concerns” and “high risk” bias lie within the “measurement of the outcome” domain, as seen in Table 1. This suggests that bias in outcome measurement across the included studies may come from factors such as unblinded outcome assessors, the use of inadequate or inappropriate measurement instruments, or selective reporting of data [48]. Potential weaknesses in the evidence are highlighted by the fact that 37.5% of the studies have a “high risk” in outcome measurement, while 43.8% raise “some concern” regarding bias. While studies with a modest concern may impact the overall direction of results, those with a high risk of bias may overstate or underestimate treatment effects. As a result, the robustness of the findings may be compromised, limiting the reliability and generalizability of the conclusions. This variability underscores the need for more standardized and rigorous methodologies to ensure greater consistency and reliability in the results.

All of the 17 articles that were included were randomized controlled trials, and they included 642 people, 864 active tDCS sessions (1–2 mA), and 108 PBM sessions of stimulation in female and male patients aged 65 to 78 (see Table 2 and Table 5). In other words, more than 900 sessions were conducted in these studies [24,49,50,51,52,53,54,55,56,57,58,59,60,62,63,64,66]. This is particularly important for understanding tDCS and PBM efficacy, as the effects of tDCS are expected to occur after multiple sessions. Both PBM and tDCS clinical treatment protocols require several sessions.

One challenge in conducting the research synthesis for this article was the heterogeneity of the included research articles. Regarding studies using the tDCS technique, varying neuroimaging techniques, electrode locations, saline solutions, and different repeated-measure time intervals were used. Some studies utilized sham, while others utilized only active tDCS [57,60]. In most studies, cognitive effects were recorded using different psychological scales. Also, adverse events or side effects were recorded with various or no scales.

Despite some of the heterogeneities, the overall tDCS across all studies remained within the standards (for tDCS, 0–2 mA, 10–20 min sessions, 1–20 sessions). One study conducted by the same authors was built upon their prior investigations [53]. Most of the studies predominantly involved participants from clinical samples. Lastly, it has been demonstrated that the polarizing effects of tDCS vary based on titrated amperage and the session duration. It remains to be seen whether these varying polarizing effects are consistent or whether they have an impact on efficacy and safety. We report studies stimulating for 10–30 min at 0–2 mA, with follow-up measures extending up to 8 months. In all studies that compared active to sham stimulation, the population in the active stimulation group showed cognitive improvements [24,51,52,53,54,55,56,58,59,62,63,66]. All protocols have demonstrated positive evidence of efficacy and safety (Table 1).

Regarding electrode location, tDCS appeared effective at all electrode sites. Future reviews targeting specific brain regions will clarify whether there are consistent differences in efficacy at different electrode sites. Electrode preparation is essential because particular solutions are more irritable. For example, water is not ideal, as it contains metals that could increase the irritability of the scalp when a current is passed through it. Communications from the 2019 International Brain Stimulation Conference [68] have suggested that a syringe should apply saline to each electrode to control the specific amount of saline content for each subject (instead of soaking the entire electrode). Henceforth, studies should report their electrode preparation. Therefore, the authors should report the electrode size, saline, and other electrode preparation materials. Although manufacturers’ materials and features may vary, the underlying method remains consistent. When both the device and electrodes are constructed from high-quality materials and are controlled correctly by trained personnel, with an amperage range from 0 to 2 mA, they should each demonstrate a comparable level of efficacy. Regarding the equipment utilized in studies included in this review, we noticed that each study used a distinct machine [51,52,53,54,55,56,57,58,59,60,61,62,63,66] that was inappropriate for an aggregate synthesis.

There is a notable lack of medication reporting in existing studies and the broader tDCS literature. In a 2018 review, McLaren [69] highlighted that the tissue excitability affected by tDCS can be altered by the medications being used, suggesting that medication may impact the efficacy of tDCS. As noted in the Introduction, pharmacological intervention is the most common therapeutic approach in the treatment of Alzheimer’s disease (AD) [18]. The use of anti-dementia medications has been shown to slow the progression of the disease potentially and, in some cases, improve cognitive functions [70]. However, the majority of the studies included in this review did not provide a comprehensive drug history for the patients. Furthermore, none of the studies specifically analyzed whether the use of anti-dementia drugs influenced the efficacy of transcranial direct current stimulation (tDCS). Many studies included in this review took a generalized approach to medication, stating that no medication was used or that participants continued their usual medication. This may be due to the assumption that pharmacological treatment for Alzheimer’s disease (AD) is a standard approach, leading the studies to focus on whether transcranial direct current stimulation (tDCS) could serve as a complementary therapeutic intervention. While this has generally led to safe outcomes, potential unsafe interactions may exist. This can only be identified through thorough and consistent reporting of medication use by participants and researchers. Following McLaren’s recommendations [69], it would be beneficial for all brain stimulation studies to include a detailed medication history during participant screening where relevant.

Considering the imaging methods used, EEG and MRI were mainly described. One study used FDG-PET scans. Two studies used imaging techniques to provide a computational model [55,61]. Since EEG is accessible and inexpensive, its combination with tDCS is expected to become more prevalent in improving treatment outcomes [51]. MRI is a valuable resource utilized in research. According to Philippen [59], tDCS may have the potential to restore a functionally impaired system and could improve hippocampus-dependent spatial memory consolidation in patients with AD. This is further supported by resting-state functional MRI analyses demonstrating restored neuronal network connectivity. Thus, similar to EEG, MRI can also be used to enhance the efficacy of including tDCS in the treatment plan.

The efficacy of tDCS could be assessed by analyzing scores from scales used to determine its results. The variability of psychological scales used to assess various cognitive abilities is considerable. However, most studies (n = 13) utilized the MMSE for evaluating global cognition [51,52,53,54,55,56,60,61,62,63,66]. Some included studies provided interpretations of the psychological assessments but did not offer comprehensive information regarding the cognitive domains evaluated through the applied scales [49,50,54,57,64,66,71]. In contrast, others provided detailed analyses of the cognitive domains evaluated to assess efficacy. The mental functions that demonstrated improvement following tDCS therapy included memory (verbal [55,56], episodic [52,53,58], spatial consolidation [59], immediate [62], and delayed [61,62]), although Jamie’s study [55] found no improvement in delayed memory. Additionally, improvements were noted in attention [52,53,60], temporal and personal orientation [52,53], calculation [52,53], linguistic abilities [55,56], verbal fluency [63], and learning ability [62]. However, after applying tDCS, no improvement was observed in immediate learning performance [59].

Many of the tests employed were modified to align with the nationality of the tested population. Although tDCS is considered to be one of the safest techniques of NIBS methods [37], the secondary outcomes of this review, namely safety and tolerability, were not as thoroughly described.

Out of the fourteen studies included in evaluating the efficacy and safety of transcranial direct current stimulation (tDCS), only six reported adverse effects [54,57,59,60,62,63]. Lane et al. [57] described the side effects of the pharmaceutical interventions used with the neuromodulation approach; however, it did not mention any negative effects associated with tDCS treatment. The most commonly reported adverse effects in the included studies were discomfort, soreness, fatigue, and a sensation of burning or heat, all of which are frequently cited in the existing literature [37]. Additionally, an uncommon side effect, such as metallic taste, was reported in one study [59]. In most cases, these symptoms described were mild. Most of the studies included in this review that reported side effects did not provide detailed information regarding the specific groups to which participants were assigned. One study that provided such information [59] found that, even when sham stimulation was applied, participants reported not only mild- but also moderate-intensity effects. This could potentially be attributed to a lower pain threshold or an increased susceptibility to placebo effects.

PBM is a technique utilized as a complementary therapy in a variety of neurological and psychiatric diseases [42]. Due to the limited number of included studies and the heterogeneity observed, it is impossible to standardize the PBM technique (Table 3). All participants in the studies on PBM therapy were drawn from clinical samples. Although there are variations in study protocols, all studies demonstrated positive efficacy evidence; however, no safety evidence was reported [49,50,64].

Nevertheless, it is essential to consider that the method of administration may influence the effectiveness of PBM. All devices used were distinct, and each technique of delivering PBM varied (transcranial, transcatheter, intranasal). The heterogeneity of the machine parameters is significant. In all the studies included, protocols varied, resulting in distinct sessions and timeline requirements [49,50,64].

The study by Maksimovich [64] provided information regarding the use of anti-dementia medication and identified which group (the control group, in this case) received it. They did not examine the interaction between medication and PBM; instead, they compared the effects of medication versus PBM. This study suggests that PBM may be more effective than anti-dementia medications, as it promotes cerebral angiogenesis, enhances hemodynamics, reduces hypoxia, and stimulates neurogenesis, ultimately leading to improved cognitive function. In contrast, anti-dementia medications may only stabilize early-stage Alzheimer’s patients temporarily without improving cognitive function, and they become ineffective in the later stages of the disease. However, the other studies included in the analysis of the PBM technique did not conduct an interaction analysis between the use of medication, PBM, and cognitive function. This omission may stem from the assumption that anti-dementia medication is considered a standard therapeutic approach, while PBM is viewed as a complementary treatment. As such, the focus of these studies may have been to explore whether PBM could offer supplementary benefits alongside the medication. Considering McLaren’s recommendations [69], it would be helpful for all PBM studies to include a comprehensive record of all medications used where relevant. While this has generally resulted in safe outcomes, potentially harmful drug interactions may still occur, which can only be identified through comprehensive and consistent reporting of medication use by both participants and researchers.

It is fair to state that the studies included in this review report inconsistent neuroimaging evidence regarding the efficacy of PBM. Future research utilizing neuroimaging to evaluate the effects of PBM should define the structural or functional markers being measured and outline their expectations.

All studies included in this review stated that PBM is efficient as a therapy for people with AD. In all studies, specific questionnaires were used, enabling patients to assess systematic changes in task performance or self-reported outcomes that showed improvement in cognitive function after PBM therapy. Regarding the secondary outcomes of this study, like safety and tolerability, limited or no information was provided. Therefore, future research is strongly encouraged.

## 5. Conclusions

In this review, we summarized 17 studies that detailed the clinical experience of tDCS and PBM in the management of treatment for individuals with AD. One could argue that the efficacy of tDCS and PBM for an adult population with AD is of global interest (see Table 2) due to the variability of the countries that analyzed these techniques (n = 11). Although minimal side effects were reported, tDCS and PBM are considered to be highly tolerable and safe. As a result, neuromodulation techniques, like tDCS and PBM, are effective in improving cognitive abilities in individuals with AD and customizing treatment options.

While tDCS and PBM have shown potential for improving cognitive function in Alzheimer’s disease, the heterogeneity of studies and the identified risk of bias must be considered when interpreting these findings. To strengthen the available evidence, future research should focus on standardizing stimulation protocols, systematically evaluating the safety of PBM, and using neurophysiological tools to measure the effects of both techniques with greater precision.

## 6. Limitations

The fact that researchers involved in this study were not blinded could have led to some selection bias. Interpreting results could also have potential bias, as reported in Section 2.4.

## Figures and Tables

**Table 1:** Risk of assessment bias for the included studies.

Reference	Randomization Process	Timing of Identification or Recruitment of Participants	Deviations from Intended Interventions	Missing Outcome Data	Measurement of the Outcome	Selection of the Reported Result	Overall Bias
Nagy et al. [49]	Low	Low	Low	High	High	Some concerns	High
Razzaghi et al. [50]	Low	Low	Low	Low	Low	Low	Low
Andrade et al. [51]	Low	Low	Low	Low	Low	Low	Low
Gangemi and Fabio [52]	Low	Low	Some concerns	Low	High	Some concerns	Some concerns
Gangemi et al. [53]	Low	Low	Low	Low	Some concerns	Low	Some concerns
Hu et al. [54]	Low	Low	Low	Low	Low	Low	Low
J.J. Im et al. [55]	Low	Low	Low	Low	Some concerns	Low	Some concerns
Kim J and Yang Y [56]	Low	Low	Low	Low	High	Low	High
H.-Y. Lane et al. [57]	Low	Low	Low	Low	High	Low	Some concerns
Li et al. [58]	Low	Low	Low	Low	Some concerns	Low	Some concerns
Philippen et al. [59]	Low	Low	Low	Low	Some concerns	Low	Some concerns
Pini et al. [60]	Low	Low	Low	Low	Low	Low	Low
Rasmussen et al. [61]	Low	Low	Low	Low	Some concerns	Low	Some concerns
Satorres et al. [62]	Low	Low	Low	Low	Some concerns	Low	Some concerns
Smirni et al. [63]	Low	Low	Low	Low	High	Some concerns	High
Maksimovichi [64]	Some concerns	Low	Low	Low	High	Some concerns	High
Wang et al. [65]	Low	Low	Low	Low	High	Low	High

**Table 2:** tDCS device characteristics and materials used. DLPFC = dorsolateral prefrontal cortex, L = left, R = right, PL = parietal lobe, rTPJ = right temporoparietal junction, RIP = right inferior parietal, DMN = default mode network, RDLPF = right dorsolateral prefrontal, and SN = salience work.

Study	Device Used	Amperage Used (mA)	Electrode Location	Electrode Size (cm)	Soaking Solution	Medication Reported
Wang et al. (2024) [65]	Neuroelectrics STARSTIM	2	DLPFC	5	Saline	None to be reported
Smirni et al. (2021) [63]	BrainStim	1	DLFPC	5	Saline	None to be reported
Satorres et al. (2023) [62]	Newronika	2	DLFPC	5	Sterile water	None to be reported
Andrade et al. (2022) [51]	TCT-Research neurostimulator	2	L, R DLPFC, Broca, Wernicke, L, R PL	5	Saline	None to be reported
Gangemi (2020) [52]	Braindee	2	DLPFC	2.5	Saline	Yes
Gangemi (2021) [53]	Braindee	2	DLPFC	2.5	Saline	Yes
Hu et al. (2022) [54]	Tianjin Timus Medical	2	Angular gyrus	None to be reported	Saline	Yes
J.J. Im et al. (2019) [55]	YDS 301	2	DLPFC	6	Saline	None to be reported
Kim and Yang (2023) [56]	YMS 201	2	DLPFC	None to be reported	Saline	None to be reported
H.-Y. Lane et al. (2023) [57]	Neurocon	2	DLPFC	5	None to be reported	Yes
Li X et al. (2023) [58]	TDCS 20A	2	DLPFC	5	None to be reported	None to be reported
Philippen et al. (2024) [59]	Tarstim 32	2	rTPJ	None to be reported	None to be reported	Yes
Pini et al. (2022) [60]	Brainstim	1.5	RIP DMN node, RDLPF SN node	None to be reported	5	None to be reported
Rassmusen et al. (2021) [61]	Starstim	2	DLPFC	1.2	None to be reported	None to be reported

**Table 3:** tDCS characteristics for population and age.

Study	Country of Study and Type of Population	Other Diagnoses Beside AD	Mean Age	Participant Number	Active /Sham	Other NIBS Methods	Efficacy	Side Effects Reported
Wang et al. (2024) [65]	Taiwan/Asian	No	75.6	30	Yes/ yes	No	Yes	No
Smirni et al. (2021) [63]	Italian/ European	No	73.17	40	Yes/ no	No	Yes	Yes
Satorres et al. (2023) [62]	Spanish/ European	No	75	33	Yes/ yes	No	Yes	Yes
Andrade et al. (2022) [51]	Brazil/ South American	No	76.3	36	Yes/ yes	No	Yes	No
Gangemi (2020) [52]	Italy/ European	No	None reported	26	Yes/ yes	No	Yes	No
Gangemi (2021) [53]	Italy/ European	No	68.6	18	Yes/ yes	No	Yes	No
Hu et al. (2022) [54]	China/ Asian	No	76.2	21	Yes/ yes	TMS	Yes	Yes
J.J. Im et al. (2019) [55]	Republic of Korea/Asian	No	73.4	20	Yes/ yes	No	Yes	No
Kim and Yang (2023) [56]	Republic of Korea/ Asian	No	70.5	16	Yes/ yes	No	Yes	No
H.-Y. Lane et al. (2023) [57]	Taiwan/ Asian	No	74.3	48	Yes/ no	No	Yes	Yes
Li X et al. (2023) [58]	China/ Asian	No	76.14	124	Yes/ yes	No	Yes	No
Philippen et al. (2024) [59]	Germany/ European	MCI	None reported (only for tDCS)	12	Yes/ yes	No	Yes	Yes
Pini et al. (2022) [60]	Italy/ European	bvFTD behavioral variant frontotemporal dementia	72.5	26	Yes/ no	No	Yes	Yes
Rassmusen et al. (2021) [61]	Norway/ European	No	78.80	19	Yes/ yes	No	Yes	No

**Table 4:** PBM device characteristics and evaluated parameters.

Study Included	Device Used/	Administration Mode	Wavelength, nm	Beam Size	Timing of Treatment/Session	Numbers of Sessions	Active /Sham	Efficacy	Side Effects Reported
Razzaghi [50]	Hoosh Yar Gamma	Transcranial	810	3.14 cm^2^	20 min	6×/week, 12 weeks	Yes/yes	Yes	None to be reported
Maksimovich [64]	Helium–neon laser ULF-01	Transcatheter Intracerebral	632.8	1–2 mm	1200–2400 s	1 time only, on both the right and left hemispheres	Yes/ no	Yes	None to be reported
Nagy [49]	LASPOT	Nasal and wrist laser acupuncture watch	650 nm	None to be reported	30 min per session	2 times/day, 3 days/week for 3 months	Yes/ yes	Yes	None to be reported

**Table 5:** PBM population demographics.

Study	Country of Study and Type of Population	Other Diagnoses Besides AD	Participant Number	Mean Age
Razzaghi et al. (2024) [50]	Iran/Asian	MCI	16	75.25
Maksimovich (2022) [64]	Russia/European	Binswanger disease	97	67.5
Nagy et al. (2021) [49]	Egypt/ African	No	60	69.75

## Data Availability

Data are contained within this article.
